# Defining Surgical Terminology and Risk for Brain Computer Interface Technologies

**DOI:** 10.3389/fnins.2021.599549

**Published:** 2021-03-26

**Authors:** Eric C. Leuthardt, Daniel W. Moran, Tim R. Mullen

**Affiliations:** ^1^Department of Biomedical Engineering, Washington University, St. Louis, MO, United States; ^2^Department of Neurological Surgery, Washington University School of Medicine, St. Louis, MO, United States; ^3^Department of Neuroscience, Washington University School of Medicine, St. Louis, MO, United States; ^4^Department of Mechanical Engineering and Materials Science, Washington University, St. Louis, MO, United States; ^5^Center for Innovation in Neuroscience and Technology, Washington University School of Medicine, St. Louis, MO, United States; ^6^Brain Laser Center, Washington University School of Medicine, St. Louis, MO, United States; ^7^Division of Neurotechnology, Washington University School of Medicine, St. Louis, MO, United States; ^8^Intheon Labs, San Diego, CA, United States

**Keywords:** brain computer interface (BCI), neuroprosthetic, surgical risk, terminology, ECOG, single neuron, local field potential, EEG

## Abstract

With the emergence of numerous brain computer interfaces (BCI), their form factors, and clinical applications the terminology to describe their clinical deployment and the associated risk has been vague. The terms “minimally invasive” or “non-invasive” have been commonly used, but the risk can vary widely based on the form factor and anatomic location. Thus, taken together, there needs to be a terminology that best accommodates the surgical footprint of a BCI and their attendant risks. This work presents a semantic framework that describes the BCI from a procedural standpoint and its attendant clinical risk profile. We propose extending the common invasive/non-invasive distinction for BCI systems to accommodate three categories in which the BCI anatomically interfaces with the patient and whether or not a surgical procedure is required for deployment: (1) *Non-invasive*—BCI components do not penetrate the body, (2) *Embedded*—components are penetrative, but not deeper than the inner table of the skull, and (3) *Intracranial* –components are located within the inner table of the skull and possibly within the brain volume. Each class has a separate risk profile that should be considered when being applied to a given clinical population. Optimally, balancing this risk profile with clinical need provides the most ethical deployment of these emerging classes of devices. As BCIs gain larger adoption, and terminology becomes standardized, having an improved, more precise language will better serve clinicians, patients, and consumers in discussing these technologies, particularly within the context of surgical procedures.

## Introduction

Brain Computer Interfaces (BCIs) continue to expand in their potential for impact. In its most simplistic form, a BCI is a computing device that supplants or augments the input and/or output of the nervous system. Historically, the classical application is to bypass a deficit by establishing “a communication system in which messages or commands do not pass through the brain normal output pathways of peripheral nerves and muscles” (Wolpaw et al., 2000). More recently, BCIs have been applied to augment existing neural functionality for improved performance in healthy humans (Arico et al., 2018). For clinical applications of BCI technology, the diversity of form factors to meet different clinical indications and surgical considerations require careful consideration with regard to their description of risk and deployment. A key distinction is whether or not a BCI requires a surgical procedure for deployment or removal. Technologies that require surgical procedures are often described as “invasive” whereas those that do not require a surgical procedure are considered “non-invasive” (Allison et al., 2010). It may be unclear, however, how “invasive” should be interpreted from a risk standpoint, particularly when using terms that convey a gradation of invasiveness.

In recent years, the terms “minimally invasive” and “minutely invasive” have been used to describe emerging and prospective categories of invasive neural interface devices. *Minimally invasive* typically characterizes devices that require a surgical procedure but have a low risk of infection or where contact or disruption of neural tissue and vasculature is minimal (Hochberg and Donoghue, 2006). This includes surgical procedures with a small footprint (e.g., a minor incision) and/or which may be distal to the brain (e.g., intracranial electrode delivery via an endovascular stent or a lumbar puncture). *Minutely invasive* is a term recently introduced by the United States Defense Advanced Research Projects Agency (DARPA) which characterizes a class of prospective devices that could be introduced to the brain without a surgical procedure (e.g., nano-scale devices that could be delivered to the brain through intravenous injection, ingestion, insufflation, or other non-surgical methods) (DARPA, 2019). However, while these terms are usually intended to convey gradations of surgical risk, a significant level of ambiguity remains regarding the appropriate definition and application of these terms. For instance, describing a procedure as minimally invasive may carry very distinct surgical morbidities. The imprecise language as it relates to invasiveness and risk may deter or otherwise influence decision making as to whether a device is appropriate for the given indication. This precision is important when the indication can range from a quadriplegic patient to a healthy consumer.

In this work we will briefly describe the major current and emerging BCI platforms with an emphasis on those requiring a surgical procedure, define risk profiles, and propose a terminology structure that we believe enables a more accurate and informative description of the invasiveness and clinical risk for BCIs that require surgical deployment.

There are several past and ongoing projects focused on the characterization and/or standardization of BCI platforms and their applications, including standardizing certain aspects of the BCI terminology. These projects include the Future Brain-Neural Computer Interface (Future-BNCI) and BNCI Horizon 2020 Initiatives, initiated by the European Commission in 2010 and 2013, respectively. Amongst its four major goals, the Future-BNCI project sought to establish clear standardized terminology for BCIs, emphasizing that “terms and definitions do matter” (Allison et al., 2010; Future BNCI Project, 2012). The initiative presented a clear distinction between “invasive” and “non-invasive” BCIs, with the definition that “invasive BCIs require surgery to implant the necessary sensors, whereas non-invasive BCIs do not.” However, they also noted that while “invasive” and “noninvasive” are most often used, other terms such as “intracranial” and “implanted” have also been interchangeably used (i.e., in lieu of “invasive”). While the invasive/non-invasive categorical distinction is widely used, the fact remains that there is still no unified consensus on the use of this or other terminology—particularly in a manner that conveys degree of clinical risk to a user for BCIs that require a surgical procedure.

The BNCI Horizon 2020 initiative built upon the Future-BNCI effort and sought to develop a roadmap for the BCI field and applications and outline the future of BCIs through 2020 and beyond (Allison et al., 2010; Brunner et al., 2015). This working group reinforced the key distinction between invasive and non-invasive BCIs with respect to the presence or absence of a surgical procedure. Of additional relevance to the present work is the BNCI working group's observation that invasive and non-invasive BCIs offer different solutions for different users, which we discuss further in the context of matching a BCI device to the clinical indication and balancing clinical risk with user need.

The IEEE SA Industry Connections working group recently released a BCI Standards Roadmap report, which includes a summary of various relevant standardization efforts (IEEE SA Industry Connections No. IC17-007, 2020). The working group identified that “there is a clear lack of standards and agreed practices for the terminology used to specify BMI systems” and that resolving this is an area of high priority for standards initiatives. Of note, IEEE Working Group P2731 has recently been established to create a standard for the Unified Terminology for Brain-Computer Interfaces.

We see the subject matter of our work as complementary to these and other standardization and road-mapping efforts. It represents our attempt to offer a semantic framework that is specifically relevant to the problem of conveying relative risk for BCIs that require surgical deployment and which may be helpful in the creation of BCI terminology standards. We must emphasize that the purpose of this paper is not to present a comprehensive review of contemporary BCI platforms or their performance characteristics, which has been extensively addressed elsewhere, including within the aforementioned roadmap reports. Further, the focus of this paper is limited primarily to BCI platforms that require a surgical procedure for deployment. As BCIs requiring surgical deployment gain larger adoption, having a more precise terminology to describe these systems will better serve clinicians, patients, and consumers in discussing these technologies and trade-offs between user need, information provided by the BCI, and clinical risk factors.

## Defining a Brain Computer Interface

The goal of a BCI is to restore or to augment neural functions by interfacing a computing device with the nervous system. A contemporary understanding of a BCI is “a system that measures central nervous system (CNS) activity and converts it into artificial output that replaces, restores, enhances, supplements, or improves natural CNS output and thereby changes the ongoing interactions between the CNS and its external or internal environment” (Wolpaw and Wolpaw, 2012). The source of signals used as input to the device can range from individual neuronal action potentials to electric or magnetic field potentials from larger cortical ensembles to hemodynamic changes associated with neuronal activity. Relevant signal features can include neuronal firing rate, quasi-oscillatory coherent activity in cortical ensembles, event-related potentials, functional connectivity, or changes in blood oxygen concentration, to name a few. Output effectors are also wide-ranging. Examples include computer cursor movement or object selection, robotic arm control, re-animation of paretic limbs, and synthesized language, amongst other applications. In contrast to control or communication, the output of a BCI may also be used to unobtrusively monitor a user's cognitive or affective states, such as attention, cognitive load, or emotional states, without requiring the user to consciously control or direct the BCI. The terms *active* and *passive* BCIs have been introduced to differentiate between these respective types of systems (Zander and Kothe, 2011).

By reversing the direction of information transfer from an output device, one can similarly develop an input device. In this scenario the device input is typically an electromagnetic stimulus delivered to the brain that modulates neuronal activity, thereby converting external information (e.g., light or sound) into an artificial sensation or perception (Normann et al., 2009; Liao et al., 2012; Gaylor et al., 2013). Alternatively, the input stimulus may be used to alter the neural system dynamics to change a cognitive state (e.g., enhance mood or attention). Input and output BCIs can be combined, such that they are “closed loop.” An example of this could be haptic input from sensors in the robotic arm of a prosthesis for tactile or proprioceptive feedback (O'Doherty et al., 2011; Flesher et al., 2016). BCIs may also be broadly characterized by their type of operation (e.g., motor, somatosensory, speech, auditory) and their neural interface modality (e.g., single unit neuron, electroencephalography, near infrared, etc.).

**Figure 1:**
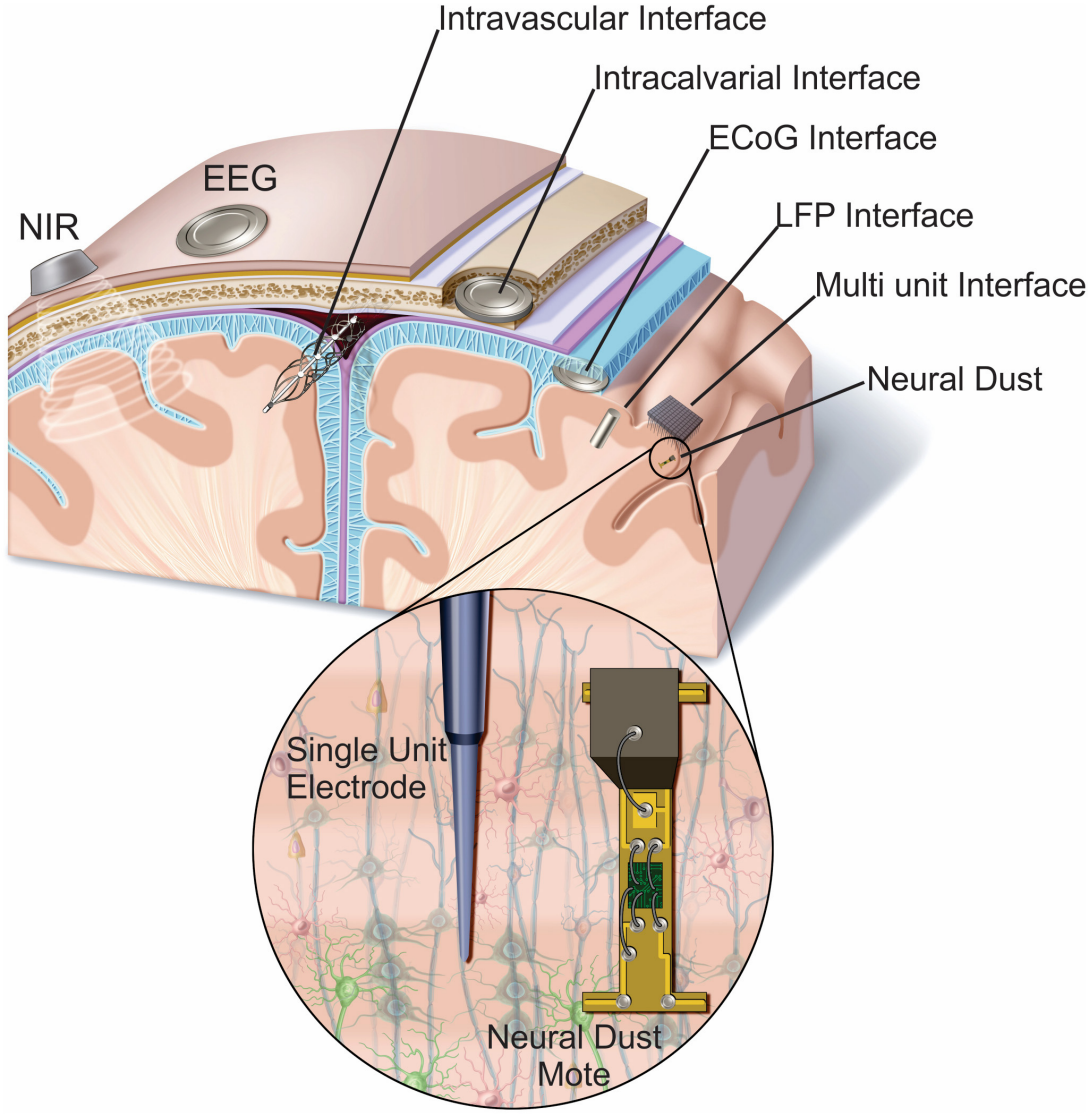
Anatomic locations of representative BCI sensors. BCI form factors have sensors in a diverse number of anatomic locations. Some are on or above the surface of the scalp (near infrared, EEG, and MEG) the others penetrate the body to varying degrees. EEG, electroencephalography; NIR, near infrared; ECoG, electrocorticography; LFP, local field potential.

In considering invasiveness and the attendant risks associated with the interface, this relies heavily on how the system physically interfaces with the patient. Thus, anatomic location requires special consideration. To this end we will briefly highlight exemplars of the different BCI platforms through this anatomic lens. Figure 1 presents a graphical summary of several of these systems. Table 1 summarizes additional characteristics, development stage, current/near-term user groups, presence/absence of catastrophic risk in deployment, and normative categorization of each system using the extended terminology proposed in this paper. For a more comprehensive examination of existing BCI platforms and their characteristics, we refer the reader to the following resources (Allison et al., 2010; Wolpaw and Wolpaw, 2012; Brunner et al., 2015; IEEE SA Industry Connections No. IC17-007, 2020). Additionally, for a detailed review of non-invasive EEG BCI systems and their current and anticipated applications, we refer the reader to a three-part series of articles published in the Special Centennial Issue of the Proceedings of the IEEE (Lance et al., 2012; Liao et al., 2012; Makeig et al., 2012).

**Table 1:** Representative current and emerging BCI systems.

**BCI modality**	**Output/Input**	**Development stage**	**Current and near-term user groups**	**Deployment catastrophic risk**	**Normative categorization**
Electroencephalography (EEG)	Output, Input	In use	Healthy users, patients, researchers	Absent	Non-invasive
Magnetoencephalography (MEG)	Output	In use	Healthy users, patients, researchers	Absent	Non-invasive
Near-Infrared (NIR) optical	Output, Input^†^	In use	Healthy users, patients, researchers	Absent	Non-invasive
Intracalvarial	Output, Input	Early stage	Potential utility for healthy users, patients, researchers	Absent	Minimally Invasive, Embedded
Intravascular	Output, Input	Early stage	Potential utility for patients, researchers	Present	Minimally Invasive, Intracranial
Local field potentials (LFPs)	Output, Input	In use	Patients, researchers	Present	Invasive, Intracranial
Microelectrodes for Single and Multi Unit Activity (SUA, MUA)	Output, Input	In use	Patients, researchers	Present	Invasive, Intracranial
Neural dust	Output, Input	Early stage	Potential utility for Patients, researchers	Present	(Minimally) Invasive, Intracranial

*Each system's development stage and current and near-term anticipated user groups are presented, alongside presence/absence of catastrophic risk in deployment and a normative categorization using the extended terminology proposed in this paper*.

† *NIR neuromodulatory effect likely via indirect mechanisms of action*.

### Electroencephalography (EEG) BCI Systems

These are BCI systems that have electrodes on the scalp which measure electric field potentials over centimeters of cortex. Due to signal transformations by the skull and scalp, EEG BCI systems are predominantly constrained to relatively high amplitude, lower-frequency brain rhythms (<90 Hz). EEG BCIs have increasingly found practical utility for passive monitoring of a wide variety of brain states (Lance et al., 2012) and for communication by tracking attention allocation and enabling control systems with 2–3 degrees of freedom (Wolpaw and McFarland, 2004; Fazel-Rezai et al., 2012; Bundy and Leuthardt, 2014). Scalp electrodes can also be used to transcranially deliver electrical current to the brain, allowing for some input control over neural activity (Paulus, 2011; Kuo and Nitsche, 2012). The low cost, low risk, maturity, and increasing wearability and diversity of applications of EEG BCI systems have led to significant adoption in both consumer and clinical applications, as well as research applications (Machado et al., 2010; Lance et al., 2012).

### Magnetoencephalography (MEG) BCI Systems

These are output-only BCI systems that measure fluctuations in magnetic fields with the same etiology as those obtained from EEG but which are unimpeded by the skull and may yield additional information regarding specific cortical activity (Waldert et al., 2008; Malmivuo, 2012; Destoky et al., 2019). The range of applications of MEG BCIs are historically similar to that of EEG, and, until recently, MEG BCIs were impractical due to the high cost and size of such systems. Recent advances in quantum magnetic field detectors which do not require superconducting technology and can be embedded in a wearable form factor is increasing the potential for practical applications in consumer and clinical sectors (Boto et al., 2018); however, it remains to be seen whether or how this will impact BCI applications.

### Near-Infrared (NIR) Optical BCI Systems

These are typically output BCI systems that measure near-infrared light to track hemodynamic activity, such as the concentration changes of oxygenated hemoglobin (HbO) and deoxygenated hemoglobin (HbR), as a local signal of brain activation (Naseer and Hong, 2015) and as inputs to a BCI. The slow hemodynamic response to transient changes in neuronal activity imposes constraints on the temporal speed of operation (Matsuyama et al., 2009) relative to electrical or magnetic sensing methods. NIR BCI systems also have the potential to function as input systems, since near-infrared and red light have been demonstrated to have potential diffuse neuromodulatory effects when applied transcranially (known as photobiomodulation), likely through an indirect mitochondrial mechanism of action as well as potentially via light-sensitive ion channels or activation of signaling mediators and transcription factors (Hamblin, 2016; Askalsky and Iosifescu, 2019). NIR BCI systems have demonstrated practical utility for healthy users as well as clinical and research applications (Naseer and Hong, 2015).

### Electrocorticography (ECoG) BCI Systems

These are neural interfaces that measure electric field potentials from the surface of the cortex with high spatiotemporal resolution (Leuthardt et al., 2009; Schalk and Leuthardt, 2011). These systems can be both input and output BCIs and have the advantage of having access to high-frequency cortical rhythms in the brain (70–300 Hz) that are associated with highly resolved information about cognitive intentions (e.g., motor kinematics and speech articulation) (Leuthardt et al., 2004, 2009; Schalk and Leuthardt, 2011; Bundy et al., 2016; Anumanchipalli et al., 2019). Current and near-term applications of ECoG BCI systems are clinical and research oriented.

### Local Field Potentials (LFP) BCI Systems

LFP BCI systems are not constrained to the cortical surface. These systems record electric field potentials from cortical populations within the brain parenchyma itself which includes cortex or deeper brain structures. Similar to ECoG, they access a broad frequency spectrum of dynamical brain activity and can be both output and input BCIs (Wang et al., 2010; Vadera et al., 2013; Li et al., 2017). Current and near-term applications of LFP BCI systems are clinical and research oriented.

### Single Unit and Multi Unit Activity (SUA, MUA) BCI Systems

These systems have microscale electrodes that are implanted within the brain parenchyma and record action potentials from single neurons. There are numerous types of electrode form factors including silicon (e.g., Utah array), microwires, carbon nanotubes, and flexible polymers (Cogan, 2008). These systems enable decoding of substantial and highly resolved information about cognitive operations (e.g., complex motor kinematics) and can be both input and output BCIs (Taylor et al., 2002; Hochberg et al., 2006, 2012; Lebedev and Nicolelis, 2006; Hatsopoulos and Donoghue, 2009; Collinger et al., 2013; Flesher et al., 2016, 2017; Musk, 2019). Current and near-term applications of these BCI systems are clinical and research oriented.

### Intravascular BCI Systems

Electrode arrays and systems that are placed within the vessels of the brain (e.g., superior sagittal sinus) to record and stimulate from adjacent brain parenchyma (e.g., motor cortex) (Watanabe et al., 2009; Oxley et al., 2016; Forsyth et al., 2019). Current and near-term applications of intravascular BCI systems are primarily clinical and research oriented.

### Neural Dust

An emerging class of millimeter-sized devices (aimed at <100 μm^3^) operated and wirelessly powered sensors/stimulators which can be used as an input/output BCI. These systems are implanted within neural tissue and each neural dust mote possesses a piezoelectric crystal that can convert mechanical power from ultrasonic pulses broadcast from outside the body into electrical power. This power can be used to study, monitor, or stimulate neural tissue and to remotely monitor neural activity (Seo et al., 2013, 2015, 2016). These systems are still in early stages of development for human intracranial use; once suitably miniaturized, applications will likely be clinical and research oriented.

### Intracalvarial BCI Systems

These are systems that are implanted but preserve the inner portion of the skull. Because of the close proximity to the cortical surface, these systems can act as input/output BCIs with capabilities similar to an ECoG BCI system but without intracranial penetration (Brodnick et al., 2019). Intracalvarial BCI systems are still nascent, but have potential applications for healthy users as well as clinical and research applications.

## Anatomic Considerations and Determination of Invasiveness

The anatomic location of the various sensor modalities plays a significant role in considering the clinical implications and risks of the systems. In broad categories, they can be divided into invasive vs. non-invasive, where “non-invasive” means that the use of the system does not require procedural insertion into the body. These types of systems would include EEG and NIR types of BCIs. Once the body is penetrated, risk considerations change with anatomic location. The next significant distinction, which applies to invasive systems, is intracranial vs. embedded. Embedded form factors include systems that are implanted in the subcutaneous, subgaleal, extracalvarial, intracalvarial, or other endo/extracranial spaces. Intracranial locations include intradural/intraparenchymal, intradural/extraparenchymal, extradural/intracranial, and intravascular locations. Complications for an invasive procedure typically include bleeding, infection, and injury to adjacent tissues. Complications in the intracranial space, however, can be substantially different from identical type of complications in embedded space. As an example, an infection in the intracranial space (e.g., meningitis, brain abscess) has the potential to be imminently life threatening and neurologically catastrophic if not addressed with a neurosurgical intervention and antibiotics. For an extracranial infection the morbidity is not as high and can likely be addressed in a less emergent fashion. This also holds true for other neurosurgical complications, such as postoperative hemorrhage. Finally, there are some anatomic locations that have unique risk considerations given their particular anatomic location. Specifically, intravascular devices, such as stent-electrodes (e.g., the Stentrode) (Oxley et al., 2016) which are placed in veins and arteries, carry the risk of perforation or occlusion which can result in a stroke or hemorrhage. While these catastrophic risks can be kept extremely low, it is important to recognize their presence, since this can have an impact in matching the form factor with the given clinical indication. This matching is essential in determining the ethical balance of risk vs. benefit, but also plays a role in clinical adoption. Even if a catastrophic risk is extremely low and the given clinical indication is appropriate, the very presence of the risk may impact a patient's decision to adopt the technology.

The term “minimally invasive” has been used frequently in describing various platforms and implicitly conflates the level of clinical risk. As an example, if a device requires a very small surgical incision that does not necessarily mean that the procedure is a lower clinical risk. If an intravascular BCI only requires a vascular access approach (2 mm puncture) is it less risky than an implantable device that doesn't penetrate the inner table of the skull, but has a larger incision? The intravascular device would carry a risk of stroke (despite the small incision), whereas a sub-scalp implant with a larger incision would not carry a concomitant stroke risk. Thus, taken together, there needs to be a terminology that best accommodates the surgical footprint of a BCI and the attendant risks with the deployment of the device.

## Extended BCI Terminology to Convey Anatomical Location and Risk—Non-Invasive, Embedded, and Intracranial

**Figure 2:**
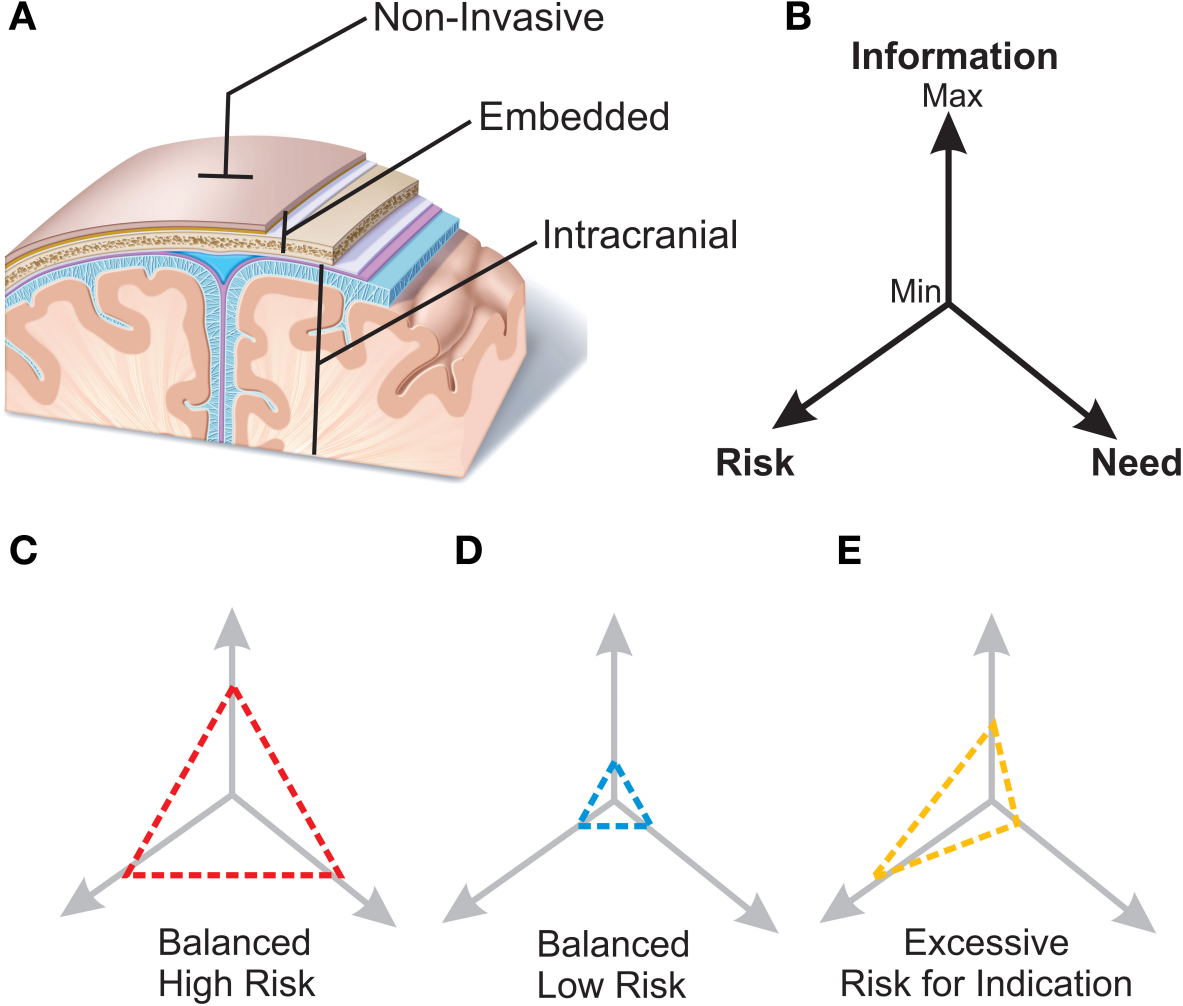
Proposed terminology for BCI systems and schematic of matching the device with the indication. **(A)** *Non-invasive*. BCI systems, such as EEG and optical based approaches, that may touch the surface of the body but do not require penetration of the skin. No surgical procedure is required. ***Embedded***. BCI systems that generally require a surgical procedure for placement, but do not enter the intracranial space. These include devices that are within the scalp, beneath the galea, or within the skull (but not through the inner table). ***Intracranial***. BCI systems that are intracranial in location and generally require a surgical procedure for placement. These include ECoG, multi-electrode arrays, neural dust, and intravascular electrode systems. **(B)** The three driving factors that influence a BCI application are clinical need, clinical risk of the device, and neurologic information necessary to support the BCI solution. Various clinical scenarios have distinct informational demands and risk tolerance. **(C)** Represents “high need—high risk” clinical indication, such as a quadriplegic patient, who has a substantial clinical need for neural restoration to improve his/her quality of life and a higher risk can be tolerated ethically. **(D)** Represents “low need—low risk” scenario where a neural interface is used for cognitive augmentation but given the low clinical need would require a lower risk to be ethically feasible. **(E)** Imbalanced scenario where information and risk are excessive to clinical need of the device.

Considering the arguments put forth in the preceding sections, we propose the following semantic framework that attempts to describe the BCI from an anatomical/risk standpoint (Figure 2).

### Non-invasive

These are BCI systems, such as EEG, MEG, and optical based approaches, that may contact the surface of the body but do not require penetration of the skin. These systems do not have any procedural risk for infection, or tissue injury. The risk for a catastrophic life threatening/altering event is absent (i.e., *catastrophic risk absent*).

### Embedded

These are invasive BCI systems that require a surgical procedure for placement, but do not enter the intracranial space. These include devices that are within the scalp, beneath the galea, or within the skull (but not through the inner table). If the device requires a surgical incision less than ~1 cm the embedded system is *minimally invasive*. These systems have procedural risk for infection or tissue injury but are not life threatening nor do they carry the risk of significant morbidity (i.e., *catastrophic risk absent*). If the system can be placed in the absence of surgical approach, we refer to the system as a *minutely invasive* embedded system. Minutely invasive embedded devices may require nanoscale techniques for deployment.

### Intracranial

These are invasive BCI systems that are intracranial in location and require a surgical procedure for placement. These include ECoG, LFPs, single units, neural dust, and intravascular electrode systems. Intracranial systems have procedural risk for infection, hemorrhage, or tissue injury which if this should occur intracranially could be life threatening and have the possibility of brain injury (i.e., *catastrophic risk present)*. Here, the terms *minimally invasive* and *minutely invasive* apply for intracranial systems in the same manner as for embedded systems.

It is important to note that these categories address the surgical description of risks associated with the placement and location of the device not only during deployment but also for surgical revision. If the system fails, becomes infected, or creates an unwanted tissue response, risk of removal must be considered. The general stratification of risks is similar as described above, but it is not an uncommon scenario that a device is relatively low risk at implantation, but the procedure for its removal is substantially more risky due to tissue adherence or need for an alternate surgical approach for explantation. Here, once again, anatomic location can play a large role. In particular, it is important to consider the durability risk. Will an implanted device continue to operate over an extended period of time? As an example, in the past there have been concerns that some microelectrodes implanted in the brain may show degraded ability to record neuronal spiking over a relatively short period of time due to astrocytic responses including glial encapsulation around electrodes (Turner et al., 1999; Suner et al., 2005; Bjornsson et al., 2006; Seymour and Kipke, 2007; Flesher et al., 2017; Salatino et al., 2017). While more recent studies have shown improved longevity in small clinical pilots (Simeral et al., 2011; Hochberg et al., 2012; Gilja et al., 2015), it will be important that the issue of durability be rigorously defined for widescale adoption. Frequent replacement of the implant could lead to local injury and would not be palatable to patients and surgeons. In this regard, invasive embedded devices (and certainly non-invasive devices) have a much lower risk than intracranial devices.

While anatomic considerations and their descriptions are essential for a surgical description of risk, there may also be contraindications which could increase the level of clinical risk for a specific BCI system. For instance, a non-invasive BCI that comes into contact with and applies pressure to the scalp could present elevated clinical risk to an individual with sensitive skin or head injury. Certain BCI system inputs could also increase risk due to underlying neurological conditions; for instance, commonly used SSVEP or P300 BCIs that present flickering stimuli may induce seizures in individuals with photosensitive epilepsy. While a thorough discussion of these and other risk factors is beyond the scope of this paper, we refer the reader to existing reports which comprehensively address this topic (Future BNCI Project, 2012; IEEE SA Industry Connections No. IC17-007, 2020).

## Matching the Device With the Indication

With the myriad form factors, anatomic locations, and potential applications of invasive BCIs, it is worth considering some general principles for how to consider a BCI and patient indication. The three driving factors that influence an invasive BCI application are (1) clinical need, (2) clinical risk of the device, and (3) neurologic information necessary to support the BCI solution. These interactions and clinical examples are highlighted in Figure 2. It is important to note that the following examples are hypothetical scenarios and do not represent the current state of technology readiness, but rather form factors and applications that emphasize these principles.

One scenario is the “high need—high risk” clinical indication (Figure 2B). As an example, a quadriplegic patient has a substantial clinical need for neural restoration to improve his/her quality of life. A robust system that could enable this type of patient to meaningfully communicate with and engage their environment (e.g., a robotic exoskeleton for walking or robotic limb capable of self-feeding) would produce substantial value to the patient. However, it would also require highly resolved information of neural intentions. Typically, higher levels of information frequently require closer proximity and more sensitive interactions with neurons and neural tissue. Put another way, increases in neural information often are parallel with the degree of invasiveness (and associated risk). In the case of a quadriplegic patient, the risk of intracranial implantation is justified to acquire the level of neural information and meaningful functional improvement.

A different example is “low need—low risk” scenario (Figure 2C) where a neural interface is used for cognitive augmentation. Essentially, a device that is enhancing neurological function beyond the normal baseline of the user. While there may be a strong demand for the technology, this “cosmetic” approach does not have a strong clinical need. Thus, the associated risk of the interface should also be low. In this case, it may not be appropriate to have an intracranial implant like one used in a quadriplegic patient where the risk of catastrophic adverse event is possible. The neurologic information and risk would be excessive relative to the intended indication (Figure 2D).

Invasive and non-invasive BCI systems convey different amounts of neural information relevant to a BCI. While the origin of activity measured by similar modality non-invasive and invasive BCIs is ostensibly the same (e.g., electrical activity measured by invasive and non-invasive BCIs originate primarily from (presynaptic) action potentials in cortical layers 1–4), non-invasive BCIs measure variants of etiologic signals which are transformed in ways that are not fully understood or may not be “reversable,” thus resulting in varying degrees of reduced information transfer relative to invasive BCI systems (Steyrl et al., 2016). In some cases, this may have little impact on BCI performance or reliability, while in other cases it can have significant effects. For instance, hand movement direction decoding accuracy and decoded information (in bits) increases progressively from EEG to MEG to ECoG to LFP to SUA (Waldert et al., 2008). While it is conceivable that future advances in physics, sensor technology, and signal processing could reverse these signal transformations, yielding an equivalence in the information conveyed by non-invasive and invasive BCIs, the field is not yet there. The cranial bone and skin tissue are primary sources of this signal transformation and associated information reduction. As such, when progressing from non-invasive, to embedded, to intracranial BCIs we may expect an increase in information conveyed by the system. Concomitant with this progression is an increase in clinical risk, albeit not necessarily in the same proportion. The relative degree of these two factors (information/risk), along with user need, should inform the decision as to whether or not a specific BCI system is appropriate in each particular situation.

The aspirational scenario is an extremely high degree of neurological information with minimal clinical risk that can be deployed across all types of need scenarios. The field is not there yet, thus a balanced approach is required when considering clinical deployment. It is important to note that the risk balance can change with time and experience. Over time, if an intracranial implant used for a high clinical need scenario has demonstrated extremely low risk experience, it can be applied to lower need-based applications. This is likely a scenario that will emerge where neural interfaces will transition from neuro-restorative approaches to those that are more focused on neural augmentation.

In addition to the appropriate balance of risk and benefit, which often guides the neurosurgeon decision to pursue an intervention, appropriately defining end-user preferences is a critical consideration both for the design and implementation of any BCI technology, but also for the success or failure of its adoption. The user may have needs and preferences for the interaction with the technology that may be distinct from the risk-benefit calculation (Lahr et al., 2015). Blabe et al. highlights this in a large survey of spinal cord injury patients (Blabe et al., 2015). Here they find that patients would prefer an unobtrusive, autonomous BCI system for both restoration of upper extremity function and control of external devices such as communication interfaces, even if the device is more invasive and higher risk than one that is aesthetically unpleasing, unreliable, or difficult or embarrassing to use. Some of these trade-offs currently are still hypothetical in that current invasive devices are not unobtrusive but may be in the future. While a detailed examination of user experience factors in BCI design is outside the scope of this paper, we refer the reader to two papers (Huggins et al., 2011; van de Laar et al., 2012) which address these factors, as well as aforementioned Future BNCI and IEEE BCI Roadmap reports.

## Conclusion

BCI form factors require a more precise language to categorize their surgical impact and risk. The commonly used two-class terminology that distinguishes between “non-invasive” (non-surgical) and “invasive” (surgical) BCIs carries significant ambiguity with respect to the surgical footprint of a BCI and the attendant risks with the deployment. We propose that having more anatomically specific descriptions—“Non-invasive,” “Embedded,” and “Intracranial”—with defined associated risk (i.e., presence or absence of catastrophic risk) will better enable the clinical, engineering, and patient community to discuss these technologies more effectively, particularly when considering tradeoffs between surgical risk, user need, and BCI informativeness/utility. We see this work as complementary to ongoing efforts to standardize BCI terminology. It represents our attempt to offer a semantic framework that is specifically relevant to the problem of conveying relative risk for BCIs that require surgical deployment and which may be helpful in the creation of BCI terminology standards.

## Data Availability Statement

The original contributions presented in the study are included in the article/supplementary material, further inquiries can be directed to the corresponding author/s.

## Author Contributions

EL conceived of the paper. EL and TM wrote the manuscript. DM edited and provided additional insights. All authors contributed to the article and approved the submitted version.

## Conflict of Interest

EL has equity in Neurolutions, Inner Cosmos, and Sora Neuroscience. TM has equity in Inner Cosmos and Intheon. DM has equity in Neurolutions and Inner Cosmos.

## References

[B1] Allison B. Millán J. del R. Nijholt A. Dunne S. Leeb R.. (2010). Future directions in Brain/Neuronal computer interaction (Future BNCI), in Asilomar BCI Meeting 2010 (Monterey, CA: Wadsworth Center), 1–2.

[B2] Anumanchipalli G. K. Chartier J. Chang E. F. (2019). Speech synthesis from neural decoding of spoken sentences. Nature 568, 493–498. 10.1038/s41586-019-1119-1 31019317 PMC9714519

[B3] Arico P. Borghini G. Di Flumeri G. Sciaraffa N. Babiloni F. (2018). Passive BCI beyond the lab: current trends and future directions. Physiol. Meas. 39:08TR02. 10.1088/1361-6579/aad57e 30039806

[B4] Askalsky P. Iosifescu V. D. (2019). Transcranial photobiomodulation for the management of depression: current perspectives. Neuropsychiatr. Dis. Treat. 15, 3255–3272. 10.2147/NDT.S188906 31819453 PMC6878920

[B5] Bjornsson C. S. Oh S. J. Al-Kofahi Y. A. Lim Y. J. Smith K. L. Turner J. N.. (2006). Effects of insertion conditions on tissue strain and vascular damage during neuroprosthetic device insertion. J. Neural Eng. 3, 196–207. 10.1088/1741-2560/3/3/002 16921203

[B6] Blabe C. H. Gilja V. Chestek C. A. Shenoy V. K. Anderson K. D. Henderson J. M. (2015). Assessment of brain–machine interfaces from the perspective of people with paralysis. J. Neural. Eng. 12:43002. 10.1088/1741-2560/12/4/043002 26169880 PMC4761228

[B7] Boto E. Holmes N. Leggett J. Roberts G. Shah V. (2018). Moving magnetoencephalography towards real-world applications with a wearable system. Nature 555, 657–661. 10.1038/nature26147 29562238 PMC6063354

[B8] Brodnick S. K. Ness J. P. Richner T. J. Thongpang S. Novello J. Hayat M.. (2019). μECoG recordings through a thinned skull. Front. Neurosci. 13:1017. 10.3389/fnins.2019.01017 31632232 PMC6779785

[B9] Brunner C. Birbaumer N. Blankertz B. Guger C. Kübler A. Mattia D.. (2015). BNCI Horizon 2020: towards a roadmap for the BCI community. Brain-Comput. Interfaces 2, 1–10. 10.1080/2326263X.2015.1008956

[B10] Bundy D. T. Leuthardt E. C. (2014). An ipsilateral, contralesional BCI in chronic stroke patients, in Brain-Computer Interface Research, eds. Guger C. Vaughan T. Allison B. (Cham: Springer Nature), 19–29. 10.1007/978-3-319-09979-8_3

[B11] Bundy D. T. Pahwa M. Szrama N. Leuthardt E. C. (2016). Decoding three-dimensional reaching movements using electrocorticographic signals in humans. J. Neural. Eng. 13:026021. 10.1088/1741-2560/13/2/026021 26902372 PMC5535759

[B12] Cogan S. F. (2008). Neural stimulation and recording electrodes. Annu. Rev. Biomed. Eng. 10, 275–309. 10.1146/annurev.bioeng.10.061807.160518 18429704

[B13] Collinger J. L. Wodlinger B. Downey J. E. Wang W. Tyler-Kabara E. C. Weber D. J.. (2013). High-performance neuroprosthetic control by an individual with tetraplegia. Lancet 381, 557–564. 10.1016/S0140-6736(12)61816-9 23253623 PMC3641862

[B14] DARPA (2019). Six Paths to the Nonsurgical Future of Brain-Machine Interfaces. Available online at: https://www.darpa.mil/news-events/2019-05-20 (accessed December 10, 2020).

[B15] Destoky F. Philippe M. Bertels J. Verhasselt M. (2019). Comparing the potential of MEG and EEG to uncover brain tracking of speech temporal envelope. Neuroimage 184, 201–213. 10.1016/j.neuroimage.2018.09.006 30205208

[B16] Fazel-Rezai R. Allison B. Z. Guger C. Sellers E. W. Kleih S. C. Kübler A. (2012). P300 brain computer interface: current challenges and emerging trends. Front. Neuroeng. 5:14. 10.3389/fneng.2012.00014 22822397 PMC3398470

[B17] Flesher S. Downey J. Collinger J. Foldes S. Weiss J. Tyler-Kabara E.. (2017). Intracortical microstimulation as a feedback source for brain-computer interface users, in Brain-Computer Interface Research, eds Guger C. Allison B. Lebedev M. (Cham: Springer), 43–54. 10.1007/978-3-319-64373-1_5

[B18] Flesher S. N. Collinger J. L. Foldes S. T. Weiss J. M. Downey J. E. Tyler-Kabara E. C.. (2016). Intracortical microstimulation of human somatosensory cortex. Sci. Transl. Med. 8:361ra141. 10.1126/scitranslmed.aaf8083 27738096

[B19] Forsyth I. A. Dunston M. Lombardi G. Rind G. S. Ronayne S. Wong Y. T.. (2019). Evaluation of a minimally invasive endovascular neural interface for decoding motor activity, in 2019 9th International IEEE/EMBS Conference on Neural Engineering (NER) (San Francisco, CA: IEEE), 750–753. 10.1109/NER.2019.8717000

[B20] Future BNCI Project (2012). Future BNCI: A Roadmap for Future Directions in Brain/Neuronal Computer Interaction. Available online at: http://bnci-horizon-2020.eu/images/bncih2020/FBNCI_Roadmap.pdf (accessed December 10, 2020).

[B21] Gaylor J. M. Raman G. Chung M. Lee J. Rao M. Lau J.. (2013). Cochlear implantation in adults: a systematic review and meta-analysis. JAMA Otolaryngol. Neck Surg. 139, 265–272. 10.1001/jamaoto.2013.1744 23429927

[B22] Gilja V. Pandarinath C. Blabe C. H. Nuyujukian P. Simeral J. D. Sarma A. A.. (2015). Clinical translation of a high-performance neural prosthesis. Nat. Med. 21:1142. 10.1038/nm.3953 26413781 PMC4805425

[B23] Hamblin M. R. (2016). Shining light on the head: Photobiomodulation for brain disorders. BBA Clin. 6, 113–124. 10.1016/j.bbacli.2016.09.002 27752476 PMC5066074

[B24] Hatsopoulos N. G. Donoghue J. P. (2009). The science of neural interface systems. Annu. Rev. Neurosci. 32, 249–266. 10.1146/annurev.neuro.051508.135241 19400719 PMC2921719

[B25] Hochberg L. R. Bacher D. Jarosiewicz B. Masse N. Y. Simeral J. D. Vogel J.. (2012). Reach and grasp by people with tetraplegia using a neurally controlled robotic arm. Nature 485, 372–375. 10.1038/nature11076 22596161 PMC3640850

[B26] Hochberg L. R. Donoghue J. P. (2006). Sensors for brain-computer interfaces. IEEE Eng. Med. Biol. Mag. 25, 32–38. 10.1109/MEMB.2006.1705745 17020197

[B27] Hochberg L. R. Serruya M. D. Friehs G. M. Mukand J. A. Saleh M. Caplan A. H.. (2006). Neuronal ensemble control of prosthetic devices by a human with tetraplegia. Nature 442, 164–171. 10.1038/nature04970 16838014

[B28] Huggins J. E. Wren P. A. Gruis K. L. (2011). What would brain-computer interface users want? Opinions and priorities of potential users with amyotrophic lateral sclerosis. Amyotroph. Lateral Scler. 12, 318–324. 10.3109/17482968.2011.572978 21534845 PMC3286341

[B29] IEEE SA Industry Connections No. IC17-007 (2020). Standards Roadmap: Neurotechnologies for Brain-Machine Interfacing. Available online at: https://standards.ieee.org/industry-connections/neurotechnologies-for-brain-machine-interfacing.html (accessed December 10, 2020).

[B30] Kuo M. F. Nitsche M. A. (2012). Effects of transcranial electrical stimulation on cognition. Clin. EEG Neurosci. 43, 192–199. 10.1177/1550059412444975 22956647

[B31] Lahr J. Schwartz C. Heimbach B. (2015). Assessment of brain-machine interfaces from the perspective of people with paralysis related content invasive brain-machine interfaces: a survey of paralyzed patients' attitudes, knowledge and methods of information retrieval. J. Neural Eng. 12:043001. 10.1088/1741-2560/12/4/043001 26169755

[B32] Lance B. J. Kerick S. E. Ries A. J. Oie K. S. McDowell K. (2012). Brain-computer interface technologies in the coming decades, in Proceedings of the IEEE (Institute of Electrical and Electronics Engineers Inc.) (San Francisco, CA), 1585–1599. 10.1109/JPROC.2012.2184830

[B33] Lebedev M. A. Nicolelis M. A. L. (2006). Brain-machine interfaces: past, present and future. Trends Neurosci. 29, 536–546. 10.1016/j.tins.2006.07.004 16859758

[B34] Leuthardt E. C. Freudenberg Z. Bundy D. Roland J. (2009). Microscale recording from human motor cortex: implications for minimally invasive electrocorticographic brain-computer interfaces. Neurosurg. Focus 27:E10. 10.3171/2009.4.FOCUS0980 PMC2875251 19569885

[B35] Leuthardt E. C. Schalk G. Wolpaw J. R. Ojemann J. G. Moran D. W. (2004). A brain-computer interface using electrocorticographic signals in humans. J. Neural. Eng. 1, 63–71. 10.1088/1741-2560/1/2/001 15876624

[B36] Li D. Han H. Xu X. Ling Z. Hong B. (2017). Minimally invasive brain computer interface for fast typing, in 2017 8th International IEEE/EMBS Conference on Neural Engineering (NER) (San Francisco, CA: IEEE), 477–480. 10.1109/NER.2017.8008393

[B37] Liao L.-D. Lin C.-T. McDowell K. Wickenden A. E. Gramann K. Jung T.-P.. (2012). Biosensor technologies for augmented brain–computer interfaces in the next decades. Proc. IEEE 100, 1553–1566. 10.1109/JPROC.2012.2184829

[B38] Machado S. Araújo F. Paes F. Velasques B. Cunha M. Budde H.. (2010). EEG-based brain-computer interfaces: an overview of basic concepts and clinical applications in neurorehabilitation. Rev. Neurosci. 21, 451–468. 10.1515/REVNEURO.2010.21.6.451 21438193

[B39] Makeig S. Kothe C. Mullen T. Bigdely-Shamlo N. Zhang Z. Kreutz-Delgado K. (2012). Evolving signal processing for brain–computer interfaces. Proc. IEEE 100, 1567–1584. 10.1109/JPROC.2012.2185009

[B40] Malmivuo J. (2012). Comparison of the Properties of EEG and MEG in Detecting the Electric Activity of the Brain. Brain Topogr. 25, 1–19. 10.1007/s10548-011-0202-1 21912974

[B41] Matsuyama H. Asama H. Otake M. (2009). Design of differential near-infrared spectroscopy based brain machine interface, in RO-MAN 2009-The 18th IEEE International Symposium on Robot and Human Interactive Communication (San Francisco, CA: IEEE), 775–780. 10.1109/ROMAN.2009.5326215

[B42] Musk E. (2019). An integrated brain-machine interface platform with thousands of channels. J. Med. Internet Res. 21:e16194. 10.2196/16194 31642810 PMC6914248

[B43] Naseer N. Hong K. S. (2015). fNIRS-based brain-computer interfaces: a review. Front. Hum. Neurosci. 9:3. 10.3389/fnhum.2015.00003 25674060 PMC4309034

[B44] Normann R. A. Greger B. A. House P. Romero S. F. Pelayo F. Fernandez E. (2009). Toward the development of a cortically based visual neuroprosthesis. J. Neural. Eng. 6:35001. 10.1088/1741-2560/6/3/035001 19458403 PMC2941645

[B45] O'Doherty J. E. Lebedev M. A. Ifft P. J. Zhuang K. Z. Shokur S. Bleuler H.. (2011). Active tactile exploration using a brain–machine–brain interface. Nature 479, 228–231. 10.1038/nature10489 21976021 PMC3236080

[B46] Oxley T. J. Opie N. L. John S. E. Rind G. S. Ronayne S. M. Wheeler T. L.. (2016). Minimally invasive endovascular stent-electrode array for high-fidelity, chronic recordings of cortical neural activity. Nat. Biotechnol. 34, 320–327. 10.1038/nbt.3428 26854476

[B47] Paulus W. (2011). Transcranial electrical stimulation (tES - tDCS; tRNS, tACS) methods. Neuropsychol. Rehabil. 21, 602–617. 10.1080/09602011.2011.557292 21819181

[B48] Salatino J. W. Ludwig K. A. Kozai T. D. Y. Purcell E. K. (2017). Glial responses to implanted electrodes in the brain. Nat. Biomed. Eng. 1, 862–877. 10.1038/s41551-017-0154-1 30505625 PMC6261524

[B49] Schalk G. Leuthardt E. C. (2011). Brain-computer interfaces using electrocorticographic signals. IEEE Rev. Biomed. Eng. 4, 140–154. 10.1109/RBME.2011.2172408 22273796

[B50] Seo D. Carmena J. M. Rabaey J. M. Alon E. Maharbiz M. M. (2013). Neural dust: an ultrasonic, low power solution for chronic brain-machine interfaces. arXiv [Preprint]:1307.2196.

[B51] Seo D. Carmena J. M. Rabaey J. M. Maharbiz M. M. Alon E. (2015). Model validation of untethered, ultrasonic neural dust motes for cortical recording. J. Neurosci. Methods 244, 114–122. 10.1016/j.jneumeth.2014.07.025 25109901

[B52] Seo D. Neely R. M. Shen K. Singhal U. Alon E. Rabaey J. M.. (2016). Wireless recording in the peripheral nervous system with ultrasonic neural dust. Neuron 91, 529–539. 10.1016/j.neuron.2016.06.034 27497221

[B53] Seymour J. P. Kipke D. R. (2007). Neural probe design for reduced tissue encapsulation in CNS. Biomaterials 28, 3594–3607. 10.1016/j.biomaterials.2007.03.024 17517431

[B54] Simeral J. D. Kim S.-P. Black M. J. Donoghue J. P. Hochberg L. R. (2011). Neural control of cursor trajectory and click by a human with tetraplegia 1000 days after implant of an intracortical microelectrode array. J. Neural. Eng. 8:25027. 10.1088/1741-2560/8/2/025027 21436513 PMC3715131

[B55] Steyrl D. Kobler R. J. Müller-Putz G. R. (2016). On similarities and differences of invasive and non-invasive electrical brain signals in brain-computer interfacing. J. Biomed. Sci. Eng. 9, 393–398. 10.4236/jbise.2016.98034

[B56] Suner S. Fellows M. R. Vargas-Irwin C. Nakata G. K. Donoghue J. P. (2005). Reliability of signals from a chronically implanted, silicon-based electrode array in non-human primate primary motor cortex. IEEE Trans. Neural. Syst. Rehabil. Eng. 13, 524–541. 10.1109/TNSRE.2005.857687 16425835

[B57] Taylor D. M. Tillery S. I. H. Schwartz A. B. (2002). Direct cortical control of 3D neuroprosthetic devices. Science 296, 1829–1832. 10.1126/science.1070291 12052948

[B58] Turner J. N. Shain W. Szarowski D. H. Andersen M. Martins S. Isaacson M.. (1999). Cerebral astrocyte response to micromachined silicon implants. Exp. Neurol. 156, 33–49. 10.1006/exnr.1998.6983 10192775

[B59] Vadera S. Marathe A. R. Gonzalez-Martinez J. Taylor D. M. (2013). Stereoelectroencephalography for continuous two-dimensional cursor control in a brain-machine interface. Neurosurg. Focus 34:E3. 10.3171/2013.3.FOCUS1373 23724837

[B60] van de Laar B. Gürkök H. Bos D. P.-O. Nijboer F. Nijholt A. (2012). Brain–computer interfaces and user experience evaluation, in Towards Practical Brain-Computer Interfaces, eds Allison B. Dunne S. Leeb R. Del R. Millán J. Nijholt A. (Berlin; Heidelberg: Springer), 223–237. 10.1007/978-3-642-29746-5_11

[B61] Waldert S. Preissl H. Demandt E. Braun C. Birbaumer N. Aertsen A.. (2008). Hand movement direction decoded from MEG and EEG. J. Neurosci. 28, 1000–1008. 10.1523/JNEUROSCI.5171-07.2008 18216207 PMC6671004

[B62] Wang W. Chan S. S. Heldman D. A. Moran D. W. (2010). Motor cortical representation of hand translation and rotation during reaching. J. Neurosci. 30, 958–962. 10.1523/JNEUROSCI.3742-09.2010 20089904 PMC6633081

[B63] Watanabe H. Takahashi H. Nakao M. Walton K. Llinás R. R. (2009). Intravascular neural interface with nanowire electrode. Electron. Commun. Japan 92, 29–37. 10.1002/ecj.10058 21572940 PMC3092556

[B64] Wolpaw J. R. Birbaumer N. Heetderks W. J. McFarland D. J. Peckham P. H. Schalk G.. (2000). Brain-computer interface technology: a review of the first international meeting. IEEE Trans. Rehabil. Eng. 8, 164–173. 10.1109/TRE.2000.847807 10896178

[B65] Wolpaw J. R. McFarland D. J. (2004). Control of a two-dimensional movement signal by a noninvasive brain-computer interface in humans. Proc. Natl. Acad. Sci. U. S. A. 101, 17849–17854. 10.1073/pnas.0403504101 15585584 PMC535103

[B66] Wolpaw J. R. Wolpaw E. W. (eds). (2012). Brain-Computer Interfaces: Principles and Practice. Oxford: Oxford University Press. 3–15. 10.1093/acprof:oso/9780195388855.001.0001

[B67] Zander T. O. Kothe C. (2011). Towards passive brain–computer interfaces: applying brain–computer interface technology to human–machine systems in general. J. Neural Eng. 8:25005. 10.1088/1741-2560/8/2/025005 21436512

